# Silver Nanoparticles Stable to Oxidation and Silver Ion Release Show Size-Dependent Toxicity In Vivo

**DOI:** 10.3390/nano11061516

**Published:** 2021-06-08

**Authors:** Brittany Cunningham, Arek M. Engstrom, Bryan J. Harper, Stacey L. Harper, Marilyn R. Mackiewicz

**Affiliations:** 1 Department of Environmental and Molecular Toxicology, Oregon State University, Corvallis, OR 97331, USA; cunningb@oregonstate.edu (B.C.); Bryan.Harper@oregonstate.edu (B.J.H.); Stacey.Harper@oregonstate.edu (S.L.H.); 2 School of Chemical, Biological, and Environmental Engineering, Oregon State University, Corvallis, OR 97331, USA; arek.engstrom@gmail.com; 3 Oregon Nanoscience and Microtechnologies Institute, Corvallis, OR 97339, USA; 4 Department of Chemistry, Oregon State University, Corvallis, OR 97331, USA

**Keywords:** lipids, silver nanoparticles, hybrid lipid-coated silver nanoparticles, silver ion dissolution, shape-control, toxicity

## Abstract

Silver nanoparticles (AgNPs) are widely used in commerce, however, the effect of their physicochemical properties on toxicity remains debatable because of the confounding presence of Ag^+^ ions. Thus, we designed a series of AgNPs that are stable to surface oxidation and Ag^+^ ion release. AgNPs were coated with a hybrid lipid membrane comprised of L-phosphatidylcholine (PC), sodium oleate (SOA), and a stoichiometric amount of hexanethiol (HT) to produce oxidant-resistant AgNPs, Ag–SOA–PC–HT. The stability of 7-month aged, 20–100 nm Ag–SOA–PC–HT NPs were assessed using UV–Vis, dynamic light scattering (DLS), and inductively coupled plasma mass spectrometry (ICP-MS), while the toxicity of the nanomaterials was assessed using a well-established, 5-day embryonic zebrafish assay at concentrations ranging from 0–12 mg/L. There was no change in the size of the AgNPs from freshly made samples or 7-month aged samples and minimal Ag^+^ ion release (<0.2%) in fishwater (FW) up to seven days. Toxicity studies revealed AgNP size- and concentration-dependent effects. Increased mortality and sublethal morphological abnormalities were observed at higher concentrations with smaller nanoparticle sizes. This study, for the first time, determined the effect of AgNP size on toxicity in the absence of Ag^+^ ions as a confounding variable.

## 1. Introduction

There are over 1400 consumer and commercial products where silver nanoparticles (AgNPs) are incorporated such as food packaging materials [1,2,3], waste-water treatment [4,5], topical ointments or wound healing gels [6,7,8,9,10], coatings on medical devices such as stents to prevent biofilm formation [11,12,13], paints, and anti-reflective coatings, and fabric cleaning chemicals [14,15,16]. The use of AgNPs in these applications predominantly rests on their superior antimicrobial properties [13,17,18,19,20,21,22]. More recent studies suggest that AgNPs can be used as antiviral agents that have been used against HIV-1 or Hepatitis B [10,23,24,25]. In addition, because silver (Ag) has a strong x-ray attenuation compared to iodinated agents used in imaging, there is significant interest in using AgNPs for x-ray computed tomography imaging and other optical imaging applications [26,27,28,29,30,31]. There is also interest in using them as drug delivery agents for cancer therapy [32,33,34] or rheumatoid arthritis therapy [35]. Consequently, it is expected that the increased production and use will lead to a significant accumulation of AgNPs in wastewater and the environment, potentially increasing exposure to humans [36,37,38]. Since AgNPs are not regulated by the Food and Drug Administration (FDA), their prevalence and potential for human exposure necessitate that we understand the full range of the potentially harmful effects of their exposure on human health. 

Precedent literature shows that the physicochemical features (size, shape, surface coating, charge) of AgNPs play a significant role in their stability, bio-uptake, toxicity, and nanoparticle–biological interactions [39,40,41,42,43,44,45,46,47,48,49,50,51,52,53,54,55,56,57,58,59,60]. Studies show that dissolved Ag^+^ and its salts in freshwater and seawater organisms [39,40] are toxic and serve as a point of reference for AgNPs that are known to undergo surface oxidation and Ag^+^ ion release. Thus far, AgNP exposure has been linked to increased reactive oxygen species (ROS) generation and DNA damage in rats [41] and cytotoxicity in mammalian cells because of impairment of mitochondrial function [42,43,44]. Furthermore, the toxicity observed at multiple biological scales is dependent on the physicochemical features of the AgNPs [45,46,47,48,49,50]. Some studies have shown that smaller diameter spheres have been associated with increased antibacterial activity by the release of Ag^+^ [20,24], increased toxicity in zebrafish embryos [51], and higher cytotoxicity in mammalian cell culture models [42] when compared to larger diameter AgNPs [52,53,54,55]. The increased dissolution in smaller diameter AgNPs may relate to the increased reactive surface area per unit mass in the smaller particles relative to those with larger diameters. In contrast, other studies have shown that larger AgNPs are more toxic [56], or that there is no association between size and toxicity [57]. Liu et al. found that the size of the AgNPs was a bigger contributor to toxicity than the surface coating [53]. When comparing shape, Ag nanoplates have been found to have even greater antibacterial activity than spheres or nanorods [58] and exhibited more toxicity in zebrafish than nanospheres and wires, which was attributed to surface defects that damage cell membranes [48]. The discrepancy in the literature has made it difficult to predict and model structure–activity relationships because it has not taken into consideration several contributing factors that can significantly impact the outcome. For example, historically, the effect of size on nanoparticle uptake and toxicity has been challenging to study because of the tendency of AgNPs to agglomerate [51]. In addition, because most AgNPs undergo surface oxidation and Ag^+^ ion dissolution, it is difficult to separate and attribute the toxic effects between AgNPs and Ag^+^, and it is possible that the toxicity of many silver nanomaterials is attributed to their release of Ag^+^ ions [49,59,60]. The presence of Ag^+^ ions is a confounding variable that adds a layer of difficulty in attempting to understand how, and to what level, the physiochemical properties (i.e., size, shape, surface chemistry) of the AgNPs contribute to this toxicity. 

While a wide variety of AgNPs have been designed for applications such as imaging and drug delivery, to date, almost all AgNPs undergo chemical and physical transformations that lead to instability and potential toxicity derived from surface oxidation and Ag^+^ release [43,44,51,61]. The ability to shield the nanoparticle surface from surface oxidation and Ag^+^ ion release is challenging as most coatings do not completely protect the AgNP surface, offering no long-term stability in environmental and biological environments that drive toxicity. Currently, the most robust options for enhancing the long-term stability of AgNPs is by surface functionalization with covalent ligands such as polyvinylpyrrolidone (PVP) [45], lipids [62], and polyethylene glycol (PEG) [63,64,65,66], thiolated PEG [67], or coating with inert inorganic substrates such as gold [63], iron [68], or silica [69,70]. More recently, Miesen et al. showed that the use of hybrid lipid-membranes can significantly enhance the stability of spherical and triangular plate AgNPs by creating a tight packing arrangement around the Ag core, shielding the surface from oxidation and Ag^+^ ion release in a range of strongly oxidizing conditions [71]. Engstrom et al. later demonstrated that when Ag^+^ ion dissolution is controlled, the hybrid lipid-coated AgNPs are more biocompatible than those coated with citrate ligands or lipid membranes that were able to undergo surface oxidation and Ag^+^ ion release [72]. When comparing spherical hybrid-lipid-coated AgNPs (10 nm) to triangular-shaped (32 nm) particles, the spherical hybrid lipid-coated AgNPs were determined to be more toxic than triangular-shaped AgNPs, suggesting that the size and shape of the AgNPs contribute to toxicity in embryonic zebrafish *(Danio rerio*) [72]. Studies with similar hybrid lipid-coated gold nanoparticles (AuNPs) of 5, 10, and 20 nm in zebrafish embryos showed rapid toxicity with 5 nm AuNPs and significant mortality occurring at concentrations ≥20 mg/L compared to the 10 nm and 20 nm AuNPs, which showed no significant mortality throughout the 5-day experiment [73]. Using model membranes and sum-frequency gain (SFG) spectroscopy, Engstrom et al. showed that 5 nm and 10 nm AuNPs can phase into the lipid monolayer with very little energetic cost; whereas 20 nm AuNPs warped the membrane conforming it to the curvature of hybrid lipid-coated AuNPs [73]. Because the 5 nm have a lower energy barrier to cross the membrane, their uptake is greater and could lead to the increased toxicity observed [73]. While the combined use of SFG spectroscopy and in vivo toxicity assays can explain the structure–property relationships, understanding the effect of a range of sizes of AgNPs of one shape is yet to be determined. 

In this study, the hybrid lipid membrane strategy was used to produce oxidant-resistant AgNPs of five different sizes, from 20 to 100 nm stable to Ag^+^ ion dissolution. Dissolution studies were performed to verify the ability of the coated nanoparticles to resist Ag^+^ release. The long-term stability of the AgNPs was assessed using UV–Vis and inductively coupled plasma mass spectroscopy (ICP-MS) for Ag^+^ ion release and changes in agglomerate size in fishwater media (FW). The toxicity of the hybrid lipid-coated AgNPs was investigated using zebrafish embryos, a common model organism for this type of toxicological assessment because of the similarities of their physiology and cellular processes to humans [74,75,76,77]. Exposure of adult and larval zebrafish to high concentrations of AgNPs is known to cause mortality, hatching delays, developmental abnormalities, neurotoxicity, genotoxicity, oxidative stress, and toxicity to gills and intestines [48,51,52,53,54,56,59,78,79]. Furthermore, their ease of reproduction, rapid development, and transparent bodies make them a great model for high-throughput screening of materials [80,81,82,83,84,85,86]. This model is ideal to rapidly assess the effect of nanoparticle size and efficiency of uptake on toxicity without the confounding effect of Ag^+^ ions.

## 2. Methods and Materials

### 2.1. Reagents

Aqueous solutions of 20, 40, 60, 80, and 100 nm citrate-capped AgNPs were purchased from NanoComposix (San Diego, CA, USA). The full characterization data of the citrate-capped AgNPs as purchased can be found in Table S1. Sodium oleate (SOA) was purchased from TCI America (Portland, OR, USA); sodium phosphate monobasic monohydrate and sodium phosphate dibasic heptahydrate were from VWR Chemicals BDH (Radnor, PA, USA); potassium cyanide (KCN) was from purchased Mallinckrodt Inc (Portland, OR, USA); L-α-Phosphatidylcholine (PC) was purchased from Avanti Polar Lipids, Inc. (Alabaster, AL, USA); hexanethiol (HT), chloroform, and TWEEN^®^ 20 were purchased from Sigma Aldrich (St. Louis, MO, USA); and nanopure water was obtained from a Milli-Q Ultra-Pure System. Ultracentrifugation was performed with a Thermo Scientific, Sorvall ST 40R (Thermofisher Scientific, Hillsboro, OR, USA) at 4700 rpm using Vivaspin ultracentrifuge concentrators with a PES membrane (Vivaspin 20, MWCO = 10 K). 

### 2.2. Preparation of Hybrid Lipid Shielded Silver Nanoparticles (Ag–SOA–PC–1HT)

The synthesis of hybrid lipid-coated AgNPs was modified from a similar procedure [71]. Briefly, in a 100 mL Erlenmeyer flask, 50 mL of each type of citrate-capped AgNPs (as received from nanoComposix.com) was allowed to stir at 400 rpm using a magnetic stir bar. To this solution, 9.4 mM SOA was added in the following volumes for each size and allowed to stir: 33.83 μL for 20 nm, 14.55 μL for 40 nm, 9.75 μL for 60 nm, 7.14 μL for 80 nm, and 5.99 μL for 100 nm. After 20 min of stirring, the resuspended PC film (0.54 mM liposomes in 10 mM sodium phosphate buffer pH 8) was added in the following volumes and allowed to stir: 323.93 μL for 20 nm, 139.29 μL for 40 nm, 93.34 μL for 60 nm, 68.37 μL for 80 nm, and 57.33 μL for 100 nm. The PC liposome was prepared using a well-established method where a solution of PC (50 μL of 0.0216 M PC in CHCl_3_) was evaporated under a stream of N_2_ as a thin film and placed under a vacuum of 12 h to remove trace organic solvents. The film was resuspended in 2 mL of 10 mM sodium phosphate buffer pH 8 and sonicated for 90 min until the solution was clear. After 40 min of stirring, 10 mM HT in ethanol was added in the following volumes and allowed to stir: 15.90 μL for 20 nm, 6.838 μL for 40 nm, 4.582 μL for 60 nm, 3.356 μL for 80 nm, and 2.814 μL for 100 nm and the solutions allowed to incubate overnight. Because of the robust surface coating on the AgNP surface, the Ag–SOA–PC–HT can be purified to remove free citrate, PC, and SOA by incubating each 1 mL of Ag–SOA–PC–HT with 10 mM of Tween^®^ 20 (7.95 mL for 20 nm, 3.42 mL for 40 nm, 2.29 mL for 60 nm, 1.68 mL for 80 nm, and 1.41 mL for 100 nm) for 30 min before purification. The Ag–SOA–PC–HT were purified by ultracentrifugation with a Thermo Scientific Sorvall ST 40R (Waltham, MA, USA) at 4700 rpm using GE Healthcare (South Plainfield, NJ, USA) ultracentrifugal concentrators with a PES membrane (Vivaspin 20, MWCO = 10 kDa) in nanopure water for five rounds at 4 min each. AgNPs were then concentrated to a final volume of 20 mL. The Ag–SOA–PC–HT concentrations were determined by ICP-MS and can be found in Table S2. Nanoparticles were then stored at 4 °C in the dark until used. 

### 2.3. UV–Visible Spectroscopy (UV–Vis) and Dynamic Light Scattering (DLS) 

Absorbance measurements were performed with an Ocean Optics USB2000 UV–Visible spectrophotometer using a 1.0 cm path length quartz cell. Dynamic light scattering (DLS), hydrodynamic diameter (HDD), and zeta potential (ZP) for AgNPs were measured at room temperature using a Malvern Zetasizer Nano (Malvern Instruments Ltd., Worcestershire, UK). Three independent suspensions were each run in triplicate to obtain the average hydrodynamic radius (HDD), size distribution based on intensity, and ZP measurements (Figure S4). The average size and standard deviation were calculated using the maximum peak values for each hydrodynamic and ZP peak value from these triplicate trials for each suspension. 

### 2.4. Inductively Coupled Plasma—Mass Spectroscopy (ICP-MS) 

ICP-MS analysis was performed using an Agilent 7700× equipped with an ASX 500 autosample (Agilent Technologies, Folsom, CA, USA) The system was operated at a radio frequency power of 1550 W, an argon plasma gas flow rate of 15 L/min, and Ar carrier gas flow rate of 0.9 L/min. Silver was measured in NoGas mode. Data were quantified using a 9-point (0.05–100 ppb (μg/kg)) calibration curve using a single-element standard (Ag, (VHG-LAGN-100, Lot # 404-0117-1)) in 1% HNO_3_/0.5% HCl. For each sample, data were acquired in triplicate and averaged. A coefficient of variance (CoV) was determined from frequent measurements of a sample containing ~10 ppb Ag. An internal standard (Sc, Ge, Bi) continuously introduced with the sample was used to correct for detector fluctuations and to monitor plasma stability. The accuracy of the calibration curve was assessed by measuring NIST reference material (water, SRM 1643f) and found to within 8% of the expected value for Ag. Recovery for the Ag single element controls was on the low side, 25% below the expected value. On the other hand, recovery for the bovine liver SRM was 16% above the expected value (one of the NIST samples measured below the detection limit).

### 2.5. Stability Studies of Hybrid Lipid-Coated AgNPs in the Presence of CN and FW Media

To determine if the surface of the AgNPs was shielded from oxidation, the well-known cyanide (CN^−^) etch test [87] was performed. A 1 mL solution of each of the Ag–SOA–PC–HT nanoparticles was incubated with 20 µL of the 307 mM KCN. The UV–Vis spectra were collected before and after the addition of KCN at a final concentration of 6 mM and the change in the O.D. and λ_max_ monitored at the following wavelengths: 392 nm for 20 nm AgNPs, 417 nm for 40 nm AgNPs, 438 nm for 60 nm AgNPs, 463 nm for 80 nm AgNPs, and 485 nm for 100 nm AgNPs. The stability of the Ag–SOA–PC–HT nanoparticles was evaluated in FW media at 6 mg Ag/L for each AgNP type. In 20 mL glass vials, 15 mL solutions of each AgNP at 6 mg Ag/L in FW media (20 nm: 2.73 mL AgNPs in 12.27 mL of FW media; 40 nm: 2.38 mL AgNPs in 12.62 mL of FW media; 60 nm: 2.18 mL AgNPs in 12.83 mL of FW media; 80 nm: 2.15 mL AgNPs and 12.85 mL of FW media; 100 nm: 1.50 mL AgNPs in 13.50 mL of FW media) were prepared and left in the dark at room temperature for one week. From these samples, 1 mL aliquots of AgNPs were retrieved and centrifuged (4700 rpm for 4 min) using a Vivaspin column with a PES membrane with an MWCO of 3 kDa (Sartorius, Otto-Brenner, Goettigen, Germany). The filtrates were collected for ICP-MS analysis at the following timepoints: upon synthesis (0 h), 1-day, 5-days, and 7-days. The UV–Vis spectra for the AgNPs were collected over time and the change in the O.D. and λ_max_ were monitored at the following wavelengths: 392 nm for 20 nm AgNPs, 417 nm for 40 nm AgNPs, 438 nm for 60 nm AgNPs, 463 nm for 80 nm AgNPs, and 485 nm for 100 nm AgNPs over the course of 0 h, 1-day, 5-days, and 7-days.

### 2.6. Zebrafish Assay

Oregon State University’s Sinnhuber Aquatic Research Laboratory (SARL) maintains wild-type tropical 5D zebrafish (*Danio rerio*) in a freshwater flow-through system under standard laboratory conditions [61]. Following a group spawn, embryos were collected and staged to ensure that they were all in the same developmental stage. At 6 hpf, pronase was used to enzymatically dechorionated the embryos [62]. When they reached 8 hpf, the dechorionated embryos were added to 200 μL of treatment solution in clear, flat-bottom 96-well plates. These treatments consisted of suspensions of AgNPs in FW ranging in concentration from 0.25 to 12 mg/L (*n* = 16 per concentration). Treatment solutions were made by diluting stock solutions of AgNPs ranging from 20–100 nm in FW. Following the addition of the embryos, the 96-well plates were incubated at 26.9 °C under a 14:10 h light–dark cycle. The embryos, and later larval fish, were assessed at two-time points (24 and 120 hpf) using a dissecting microscope. At 24 hpf, embryos were observed for mortality, developmental progression, and the presence of spontaneous movement. They were again observed at 120 hpf. At this point, the larval fish were assessed for a series of endpoints including abnormalities of the body axis, brain, eye, caudal fin, pectoral fin, jaw, otic, pigment, snout, trunk, circulation, and somites as well as edema of the yolk sack and pericardium, and changes in the behavioral response to touch. All experiments were performed in compliance with national care and use guidelines and approved by the Institutional Animal Care and Use Committee (IACUC) at Oregon State University. 

### 2.7. Statistical Analysis

Statistical analyses were performed using RStudio Version 1.0.153 (RStudio, Boston, MA, USA). Fisher’s exact test was used to compare specific developmental endpoints between treatment and controls in the embryonic zebrafish assay. Differences were considered statistically significant at *p* ≤ 0.05.

### 2.8. Zebrafish Ag Uptake Quantification

The following analysis was performed by the USR Elemental Analysis Core. Using an Agilent 7700× equipped with an ASX 500 autosample (Agilent Technologies, Folsom, CA, USA) ICP-MS, they quantified the concentration of silver (Ag) in a subset of zebrafish taken from the toxicity assay. At the end of the 120 hpf exposure detailed above, five fish from each size treatment were removed from their wells, rinsed 3× with MQ water before being digested for ICP-MS analysis. The samples consisted of 25 zebrafish, five exposed to each of the five AgNP sizes and the control, unexposed fish. Additionally, they assessed the concentration of Ag in the FW. Controls were prepared in duplicates.

### 2.9. Sample Digestion

For digestion, the water for each sample was carefully pipetted off the fish to reduce the total digestion volume. Volumes for both fractions were measured. The buffer was set aside, the fraction containing fish was digested with 200 μL of concentrated HNO_3_ (trace metal grade, Fisher) each in Sarstedt poly-propylene culture tubes (55.516 series). The tubes were loosely capped and digested for 90 min at 90 °C using a heating block. After 24 h, the volume of each sample and initial dilution was determined. 

### 2.10. Sample Dilutions 

All sample, control, and standards were prepared and measured in 1% HNO_3_/0.5% HCl. All samples and controls were diluted by volume. Weights were also recorded but the final dilution factors were calculated and results are reported by volume dilutions. Digested samples were transferred to 15 mL metal-free polypropylene tubes (VWR 89049-170 series) and 100 μL of sample was added to 1000 μL 1% HNO_3_/0.5% HCl). All samples were vortexed before dilution and again before ICP-MS measurement. The removed buffer samples as well as the FW were diluted 2× (500 μL sample + 500 μL 1% HNO_3_/0.5% HCl). 

## 3. Results and Discussion

### 3.1. Preparation of Hybrid Lipid-Coated AgNPs of Varying Sizes

**Figure 1:**
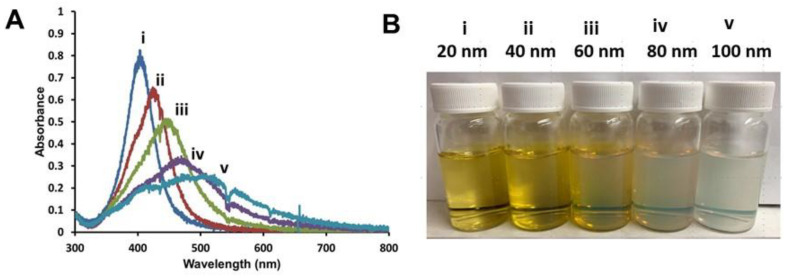
(**A**) Representative UV–Vis spectra of purified spherical Ag–SOA–PC–HT at the same concentration (6 mg Ag/L) and (**B**) photographs of (**i**) 20 nm, (**ii**) 40 nm, (**iii**) = 60 nm, (**iv**) 80 nm, and (**v**) 100 nm nanoparticles in water.

Using a well-established procedure developed by Miesen et al. to anchor membranes to AgNPs surfaces [71], a series of commercially available spherical AgNPs with diameters ranging from 20–100 nm (Nanocomposix, San Diego, CA, USA) were coated with hybrid lipid membranes comprised of a mixture of SOA, PC, and HT to yield Ag–SOA–PC–HT stabilized nanoparticles [71,72,73,88,89]. The amounts of SOA, PC, and HT used to coat the varying size of AgNPs (20–100 nm) were adjusted based on the diameter of each nanoparticle type. The combination of these surface ligands creates a complex hybrid lipid-membrane architecture that unequivocally protects AgNP from surface oxidation and Ag^+^ ion release in the presence of strong oxidants such as KCN even in the presence of membrane disrupting surfactants [71,72]. After encapsulating and anchoring the membrane with HT, Tween^®^ 20 was added to the AgNP solution to disrupt “nanoparticle-free liposomes” followed by purification by ultracentrifugation with a PES membrane with MWCO of 10 kD with nanopure water. Purification of the Ag–SOA–PC–HT is necessary to remove any excess unreacted Ag^+^ ions, lipids, SOA, or HT present that could contribute to toxicity. The 20 nm Ag–SOA–PC–HT shows a narrow-localized surface plasmon resonance (LSPR) band at λ_max_ of 404 nm (Figure 1A), measured at 6 mg Ag/L concentration. When compared to similar concentrations of AgNPs of increasing sizes, there was a prominent red-shift of the LSPR band and a decrease in the optical density (O.D.) from 40, 60, 80, and 100 nm nanoparticles with λ_max_ of 424, 446, 467, and 493 nm, respectively (Figure 1A). The increasing red-shift is consistent with the literature on the effect of increasing size and colloidal suspensions appearing cloudy (Figure 1B) [90,91]. 

### 3.2. Stability Studies of Shielded AgNPs

**Figure 2:**
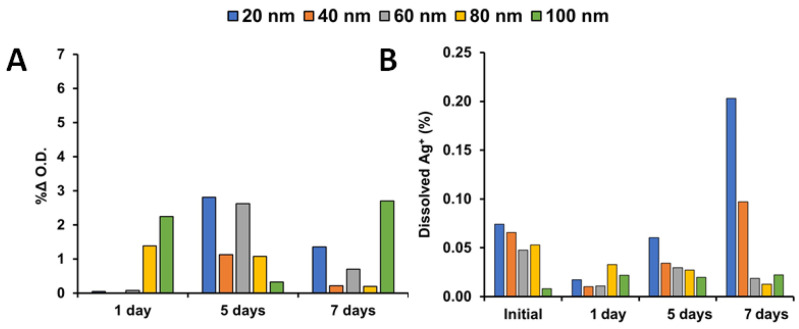
Representative |% change| in the (**A**) O.D. of Ag–SOA–PC–HT nanoparticles (20–100 nm) and (**B**) ICP-MS analysis of Ag^+^ ion release from 1 mL samples of Ag–SOA–PC–HT samples (20–100 nm) at 1-day, 5-days, and 7-days post-synthesis in FW.

The unique hybrid lipid-coating surrounding the Ag–SOA–PC–HT that prevents surface oxidative dissolution is important because it allows us to remove Ag^+^ ions as a confounding contributor in toxicological investigations and enhances the potential applications of these novel engineered nanoparticles. It is expected that the Ag–SOA–PC–HT nanoparticles will be completely shielded from surface oxidation and Ag^+^ ion release even when exposed to strong etchants such as cyanide (CN^−^) [71,72,88,89,92,93]. To ensure that each batch of Ag–SOA–PC–HT (20–100 nm) was completely coated with a hybrid lipid membrane, the AgNPs underwent a well-known CN^−^ etch test. Briefly, KCN (6 mM) was incubated with a 1 mL sample of each of the five sizes of AgNPs and UV–Vis spectra recorded before and after its addition. No significant shift in the LSPR band or decrease in the O.D. was observed after the addition of CN^−^, indicating that the AgNP surface was covered around the Ag core in a tight packing arrangement to prevent surface oxidation and Ag^+^ ion release for all sizes (Figure S1). This is consistent with previous studies of hybrid lipid-coated AuNPs and AgNPs [71,72,73,88,89]. To further determine the stability of the Ag–SOA–PC–HT nanoparticles, samples were also exposed to FW media in the dark at room temperature and the UV–Vis spectra recorded over one, five, and seven days. The UV–Vis spectra showed no significant change O.D. over time (Figure 2A) or in the LSPR band (Figure S2) and no loss of color of the AgNPs was observed over time (Figure S3). The lack of change in the UV–Vis spectra indicates that purified AgNPs are stable over seven days and even seven months with negligible Ag^+^ ion release or aggregation that could lead to a change in size impacting toxicity studies. 

Inductively coupled plasma mass spectroscopy (ICP-MS) confirmed that minimal Ag^+^ ions were released over the seven days in FW for all the sizes at the same concentration (Figure 2B). Aliquots of 1 mL samples of the varying sizes of Ag–SOA–PC–HT were filtered using a Vivaspin column with PES membrane and MWCO of 3 kDa to separate the AgNPs from Ag^+^ ions and the filtrates analyzed by ICP-MS. The amount of Ag^+^ ions released was negligible over five days, the length of time used for the toxicity assay. The 20 nm AgNPs showed a slightly higher amount of Ag^+^ after seven days followed by the 40 nm Ag–SOA–PC–HT. This indicates that the AgNPs were extremely stable in the FW with negligible or no dissolution at all sizes (Figure 2B). This is consistent with hybrid lipid bilayer coating methodologies, where SOA–PC–HT offers the most protection to the AgNPs [71,72]. Although there is precedence that shows smaller diameter AgNPs undergo Ag^+^ ion dissolution in uncoated AgNPs [94], it is unlikely that the Ag^+^ ions were present in the hybrid lipid-coated AgNP samples since there was almost no change in the UV–Vis spectra that would suggest Ag^+^ ion dissolution or a change in diameter (Figure 2A and Figure S2A). It is more likely that a AgNP passed through the PES membrane filter into the filtrate. Regardless, the stability studies showed that hybrid lipid-coated AgNPs were very stable and had minimal aggregation in the FW media used for the toxicity studies [71,72,73]. 

### 3.3. Toxicity Testing

There are currently two internationally standardized methods that leverage the benefits of the embryonic zebrafish model to define acute toxicity, OECD Test No. 236: Fish Embryo Acute Toxicity (FET) Test and ISO/TS 22082:2020: Nanotechnologies—Assessment of nanomaterial toxicity using dechorionated zebrafish embryo. While both standard assays utilize embryonic zebrafish (*Danio rerio*), there are differences in the approaches as well as the information gained from the two. The FET test is designed to provide information on the acute toxicity of chemicals on embryonic stages of fish and thus would be considered an ecotoxicological evaluation. The chorionic membrane, the outermost acellular envelope around the developing embryo, is left intact during the exposures and can form a molecular barrier that can slow or prevent the contaminant of interest from reaching the embryo. The test is run in 24-well plates with 2.5–5 mL of exposure solution for 96 h. Hatching success can be determined as most embryos will hatch between 72 and 96 h post-fertilization (hpf). Observations are made daily to identify indicators of lethality (i.e., coagulation of the fertilized eggs or lack of somite formation, tail-bud detachment from the yolk sac, and heartbeat (the heart begins beating at 24 hpf)). If any of these indicators are noted, then an LC_50_ is calculated. This study selected the ISO embryonic zebrafish assay because it (i) provides information on potential hazards of nanomaterials for other vertebrate systems including humans, (ii) minimizes the waste generated from nanotoxicity testing, and (iii) allows for the evaluation of lethal and sublethal impacts over a longer period of development. In this assay, the chorionic membrane is enzymatically removed to allow for the free movement of chemicals or nanomaterials to the developing embryo to assess a nanomaterial–biological interaction. The assay is conducted in 96-well plates with volumes of 100–200 µL, thus reducing waste generation. Embryos are exposed from 8–120 hpf, covering the periods of gastrulation to the completion of organogenesis. Observations are made at 24 hpf to assess developmental progression, mortality, spontaneous movement (one of the first behaviors of embryonic zebrafish), and notochord development. At 120 hpf, embryos are evaluated for mortality, along with malformations of the snout, brain, pectoral and caudal fins, eyes, jaw, otic structures, axis, trunk, somites, swim bladder, and body pigmentation. Physiological and behavioral endpoints are also assessed at 120 hpf including the presence of any pericardial or yolk-sac edema, impaired circulation, and impaired touch response. Thus, both an LC_50_ and EC_50_ can be calculated for mortality or sublethal effects, respectively. These advantages and the relevance to human health were the basis for using the ISO methodology instead of the OECD FET test.

**Figure 3:**
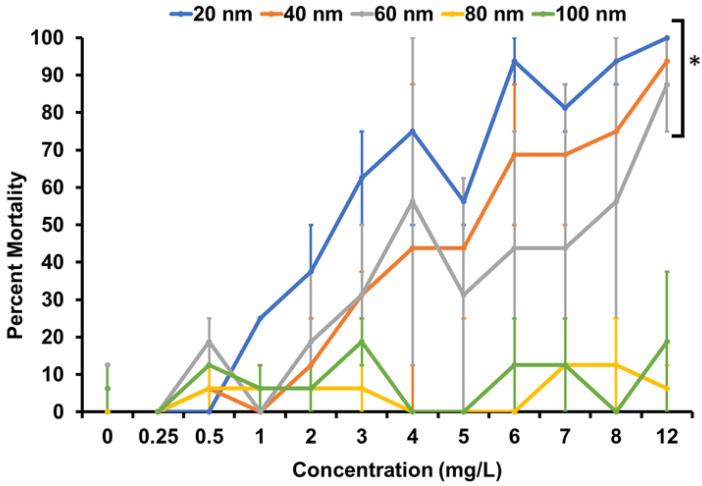
Concentration-response of zebrafish exposed to varying concentrations of hybrid lipid-coated AgNPs for total mortality at 120 hpf. Data represent two experimental replicates (*n* = 8 for each) for a total of *n* = 16 for each exposure condition. Bars show standard error. * indicates significant difference from the control (*p*-value ≤ 0.05).

To investigate the toxicity of the various sizes of AgNPs, dechorionated embryonic zebrafish were exposed to nanoparticles of either 20, 40, 60, 80, or 100 nm in size at concentrations ranging from 0.25 to 12 mg/L in FW. Ag–SOA–PC–HT were stable in FW as indicated by the lack of change in the hydrodynamic diameter (HDD) and the zeta potential in FW (Figures S4 and S5) and by lack of change in the UV–Vis spectra in the λ_max_ and O.D. that would indicate aggregation (Figure S2). Zebrafish were observed at 24 and 120 hpf and evaluated for a suite of morphological and behavioral abnormalities. In general, mortality increased with increasing concentration and decreasing size of the AgNPs (Figure 3). The greatest mortality was observed in fish exposed to both the 20 and 40 nm nanoparticles, with significant mortality starting at 3 mg/L. Additionally, the 20 nm AgNPs had 100% mortality at 12 mg/L. For the 20 and 40 nm AgNP exposures, most of the mortality (>75%) was observed at the 24 hpf timepoint. In fact, for the exposures to all nanoparticle sizes, 76.5% of the mortality observed occurred by 24 hpf. Zebrafish exposed to 60 nm AgNPs showed significant mortality beginning at 4 mg/L. Exposure to the two largest particle sizes, 80 and 100 nm, did not result in significant mortality (Figure 3). Overall, this is consistent with the literature, which shows that nanoparticle toxicity in zebrafish is size-dependent, with smaller nanoparticles being more toxic [29,34,35]. The toxicity observed in the studies with shielded AgNPs eliminates the contribution of Ag^+^ to toxicity. While it is possible that the organic hybrid-lipid membrane coating shielding the AgNPs could reasonably be a driver of toxicity, this possibility was eliminated. First, the same organic layer was in contact with the biological entity for every size of AgNP. If the organic layer is contributing to toxicity, then as the nanoparticle concentration increases for all nanoparticle types, increased toxicity would be expected as the concentration of the ligand’s present increases. However, as seen with the 80 and 100 nm, where increasing exposure concentrations of ligands are present, toxicity was not observed (Figure 3). Furthermore, the hybrid lipid-coated AgNPs had a membrane that is tightly packed around the AgNP core for each nanoparticle type and was stable even in the presence of membrane disrupting surfactants such as Tween^®^ 20 and Triton-X100, where the membrane integrity holds and ligands do dissociate from the surface that would contribute to toxicity [71]. Previous toxicity studies with 10 nm spherical AgNPs with the same membrane composition in their purified and unpurified states with excess components of liposomes, thiols, and sodium oleate showed very minimal toxicity with unpurified hybrid lipid-coated AgNPs [72]. Finally, we have conducted studies with AuNPs in the core and a hybrid lipid membrane coating, which showed 5 nm to be more toxic than 20 nm at 12 mg/L [73]. These studies indicate that the individual organic layer components do not play a role in the observed toxicity and irrelevant of the metal core composition size is a primary driver of toxicity.

In addition to increased mortality, the AgNPs also caused increases in sublethal malformations of the zebrafish in a size-dependent manner (Table 1). We observed an inverse relationship in which increasing nanoparticle size resulted in decreased sublethal effects and at increased concentrations. Exposures to AgNPs of 20, 40, 60, 80, and 100 nm resulted in significant malformations in 15, 9, 6, 1, and 0 of the morphological and behavioral endpoints, respectively. The most common observations seen in significant numbers of fish exposed to the 20, 40, or 60 nm AgNPs was a lack of spontaneous movement (SM), presence of yolk sac edema (YSE), pericardial edema (PE), and malformations of the eye, snout, and jaw. Furthermore, YSE was seen in exposures of all particle sizes except 100 nm. Fisher’s exact test (*p ≤* 0.05) was calculated to identify combinations of particle size and concentration where a significant number of zebrafish exhibited a specific abnormality; the lowest concentrations of which are termed the lowest observable adverse effect levels (LOAEL). Table 1 shows increasing LOAELs as particle size increased with no significant sublethal malformations for exposures to the largest 100 nm AgNP size. 

Many of the sublethal malformations observed have been described in the literature. For example, morphological and neurological defects are common at high concentrations of AgNPs [31,35]. AgNPs have been shown to accumulate in the tissue of zebrafish (gills, intestines, muscles [34], and head [35]), with smaller nanoparticles causing greater sublethal toxicity in the gills and intestines than larger nanoparticles [34]. For uncoated AgNPs, smaller nanoparticles are internalized more rapidly than larger nanoparticles [35]. Other published abnormalities of zebrafish exposed to AgNPs are in line with our data and include body axes malformation and cardiac effects [28], edema of the pericardium, and yolk sack [28,51], and jaw malformations [29]. Additionally, AgNPs have been shown to affect swimming behavior through neurotoxicity [59], which could explain the lack of spontaneous movement and touch response we observed. The hybrid lipid-coated AgNPs elicited similar malformations to those observed in zebrafish exposed to uncoated nanoparticles where Ag^+^ ions are thought to be the predominant driver of toxicity. However, in this case, we confirmed with hybrid lipid-coated AgNPs that the predominant driver of toxicity is the size of the nanoparticles.

This study shows that even coated AgNPs, particularly those of a smaller diameter, resulted in both lethal and sublethal effects on zebrafish. For a scenario such as this, Harper et al. (2015) proposed an embryonic zebrafish metric (EZ metric) that assigns weights to the impacts of the malformations, allowing for comparison of the mortality and morbidity observed in embryonic zebrafish development [37]. Under this metric, mortality at 24 hpf is weighted at 1.0, mortality at 120 hpf is weighted at 0.95, PE is weighted as 0.12, YSE is weighted as 0.1, lack of spontaneous movement is weighted at 0.04, and malformations to the eye, snout, and jaw are weighted as 0.02, 0.04, and 0.04, respectively. The other malformations, observed in embryos exposed to 20 nm AgNPs range in weight from 0.08–0.02 [37]. Using the calculated values as means of toxicity comparison between the AgNP sizes showed a general increase in toxicity as the size of the particle decreased (Table 1). The toxicity of AgNPs is generally attributed to their dissolution to Ag^+^ [44,45,60]. However, this study prevented dissolution to isolate effects related to the size of the AgNPs. Our study demonstrates a pattern of decreasing toxicity with increasing size of the AgNPs, indicating that, with dissolution prevented, particle size remains an important driver of toxicity. Similarly, Liu et al. (2019) noted that size is a bigger contributor to toxicity than surface coating [34]. However, the stability of coatings used in past research has been much less robust, with toxicity being attributed to changes in stability and aggregation over time [34]. In contrast, this study’s coated particles were found to be stable, with negligible dissolution or aggregation beyond the timeframe of the zebrafish exposures. Therefore, we can draw a clearer link between nanoparticle size and toxicity than has been possible in other research.

### 3.4. Quantifying Ag Uptake in Zebrafish

A subsample of the control and AgNP exposed zebrafish were taken from the toxicity assay and ICP-MS was used to assess the concentration of Ag they contained. The zebrafish samples had all been exposed to the same concentration of AgNPs in each of their respective sizes. The amount of Ag in the FW and control fish were minimal. All zebrafish exposed to AgNPs were found to have concentrations of Ag on the same order of magnitude (Table 2). These data verify that zebrafish exposed to all sizes of the coated AgNPs, 20–100 nm, internalized above-normal levels of Ag. It suggests that uptake of the coated AgNPs is size-dependent, with a greater number of 20 nm particles taken in to achieve a comparable mass to larger diameter particle uptake. Additionally, the largest AgNP, 100 nm, had the lowest uptake, which may suggest that nanoparticles of increasing size are internalized less. This would be in line with the toxicity data that showed larger nanoparticles are less toxic to zebrafish, though further research is necessary to verify this hypothesis. It should also be noted that 120 hpf zebrafish embryos were not feeding, so any uptake would be through dermal or gill adsorption coupled with incidental mouth-gaping behavior. 

## 4. Conclusions

In this study, the toxicity of AgNPs with a SOA–PC–HT surface coating was evaluated. The hybrid lipid-coating blocked AgNP dissolution, thereby removing Ag^+^ ions as a confounding contributor to toxicity. The study demonstrated the stability of these nanoparticles over time and verified that their dissolution was minimal. Past research using uncoated AgNPs has attributed the toxicity of these nanoparticles to their release of Ag^+^. However, this research has found that even when dissolution is prevented, AgNPs continue to exhibit toxicity to embryonic zebrafish. It was found that the toxicity of the SOA–PC–HT AgNPs was both concentration and size-dependent, with smaller sizes and higher concentrations being more toxic. Furthermore, the study verified that the exposed zebrafish were indeed taking up the AgNPs by measuring higher-than-normal concentrations of Ag in them. The continued use of such coated nanoparticles in toxicity studies allows for a variety of other physiochemical properties to be individually assessed without the presence of Ag^+^. Studies such as these play an important role in continuing to inform risk assessment development. Additionally, because of the important role that AgNPs play in products, particularly in the medical field, their use is unlikely to decrease any time soon. Therefore, it is important to identify the forms of AgNPs that offer the lowest levels of toxicity including reduced levels of Ag^+^ release.

## Figures and Tables

**Table 1:** Sublethal of hybrid lipid-coated AgNPs using an embryonic zebrafish model.

Lowest Observable Adverse Effect Level (mg/L)																
	24 hpf										120 hpf					
AgNP Size	DP	SM	YSE	Axis	Eye	Snout	Jaw	Otic	PE	Brain	Somite	Pectoral Fin	Caudal Fin	Circulation	Trunk	TR
20 nm	5	4	5	4	4	4	4	7	4	7	7	4	4	5	4	-
40 nm	-	8	7	-	6	6	7	-	7	-	-	7	7	-	-	7
60 nm	-	12	12	-	8	8	8	-	7	-	-	-	-	-	-	-
80 nm	-	-	8	-	-	-	-	-	-	-	-	-	-	-	-	-
100 nm	-	-	-	-	-	-	-	-	-	-	-	-	-	-	-	-

Sublethal effects of AgNPs series observed at 24 and 120 hpf indicate the significant occurrence of abnormal development. These data represent two experimental replicates of *n* = 8 for a total of *n* = 16 for each exposure condition. Legend: developmental progression at 24 hpf (DP); lack of spontaneous movement at 24 hpf (SM); 120 hpf malformations of the body axis (axis), eye, snout, jaw, otic vesicle (otic), brain, somite, pectoral fin, caudal fin, trunk, and circulation; the presence of pericardial edema (PE) and yolk sack edema (YSE); or lack of touch response (TR).

**Table 2:** Summary of Ag uptake into zebrafish and toxicity for AgNPs used to assess risk to zebrafish.

Sample	Ag	LOAEL
Fishwater	0.18 ng per ml	NA
Control Fish	0.078 ng per fish	NA
20 nm AgNP	1.478 ng per fish	3 mg/L
40 nm AgNP	1.972 ng per fish	3 mg/L
60 nm AgNP	0.62 ng per fish	4 mg/L
80 nm AgNP	1.244 ng per fish	8 mg/L
100 nm AgNP	0.356 ng per fish	-

## Data Availability

Data are contained within the article and Supplementary Material.

## Supplementary Materials

The following are available online at https://www.mdpi.com/article/10.3390/nano11061516/s1, Figure S1: Representative UV-vis spectra of purified spherical Ag-SOA-PC-HTs, Figure S2: Representative change in the O.D. of Ag-SOA-PC-HT nanoparticles in FW, Figure S3: Photographs of Ag-SOA-PC-HT nanoparticles in FW, Figure S4: Hydrodyanmic diameter and Zeta potential of Ag-SOA-PC-HT nanoparticles in FW, Table S1: Properties of citrate-capped AgNPs, Table S2: Ag-SOA-PC-HT concentrations determined by ICP-MS, Figure S5: Representative size distrubtion intensity curves.
