# Supplementary material for: Silver Nanoparticles Stable to Oxidation and Silver Ion Release Show Size-Dependent Toxicity In Vivo
Source: Nanomaterials (Basel). 2021 Jun 8;11(6):1516. doi: 10.3390/nano11061516 (PMC8230025; doi:10.3390/nano11061516)
Supplement: Supplementary file 1 [file nanomaterials-11-01516-s001.zip › nanomaterials-1225632-supplementary.pdf]

nanomaterials

MDPI

Article

Silver Nanoparticles Stable to Oxidation and Silver Ion Release Show Size-Dependent Toxicity In Vivo

Brittany Cunningham [1], Arek E. Engstrom [2], Bryan J. Harper [1], Stacey L. Harper [1,2,3] and Marilyn R. Mackiewicz [4,*]

[1] Department of Environmental and Molecular Toxicology, Oregon State University, Corvallis, OR, 97331 USA; cunningb@oregonstate.edu (B.C.); Bryan.Harper@oregonstate.edu (B.J.H.); Stacey.Harper@oregonstate.edu (S.L.H.)
[2] School of Chemical, Biological, and Environmental Engineering, Oregon State University, Corvallis, OR, 97331 USA; arek.engstrom@gmail.com
[3] Oregon Nanoscience and Microtechnologies Institute, Corvallis, OR, 97339 USA
[4] Department of Chemistry, Oregon State University, Corvallis, OR, 97331 USA
* Correspondence: marilyn.mackiewicz@oregonstate.edu

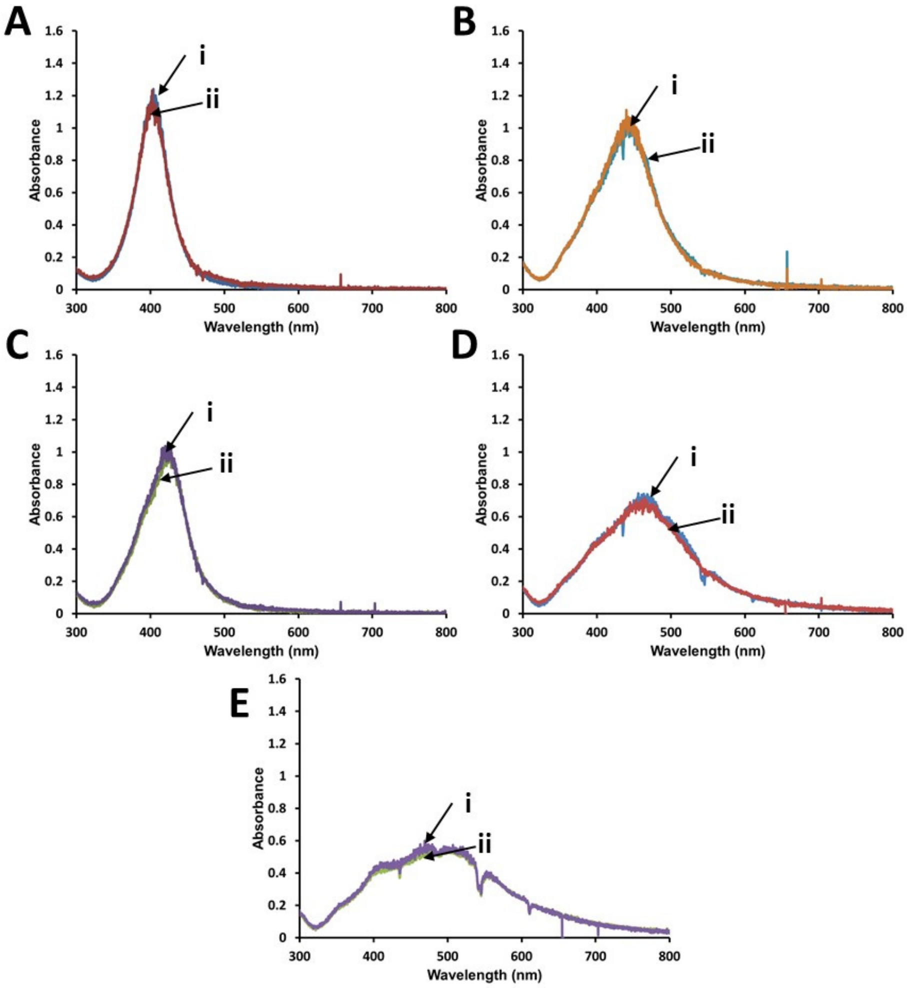

Figure S1. Representative UV-vis spectra of purified spherical Ag-SOA-PC-HT at the same concentration (6 mg Ag/L). **A)** 20 nm, **B)** 40 nm, **C**) = 60 nm, **D**) 80 nm, and **E**) 100 nm nanoparticles i) before and ii) after exposure to KCN (6 mM) in water.

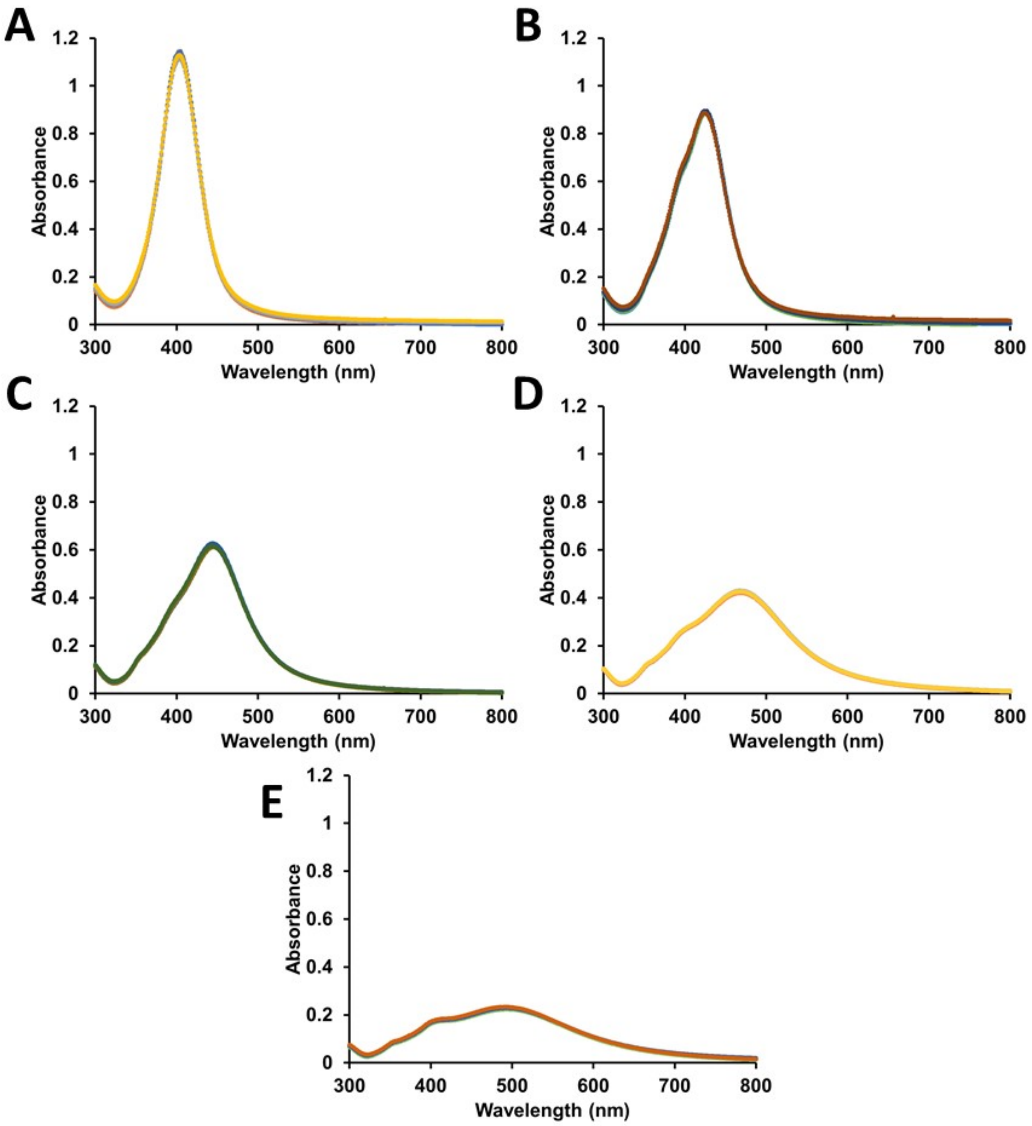

Figure S2. Representative |% change| in the O.D. of Ag-SOA-PC-HT nanoparticles **A)** 20 nm, **B)** 40 nm, **C**) = 60 nm, **D**) 80 nm, and **E**) 100 nm nanoparticles at 1-day, 5 days, and 7 days post synthesis in FW.

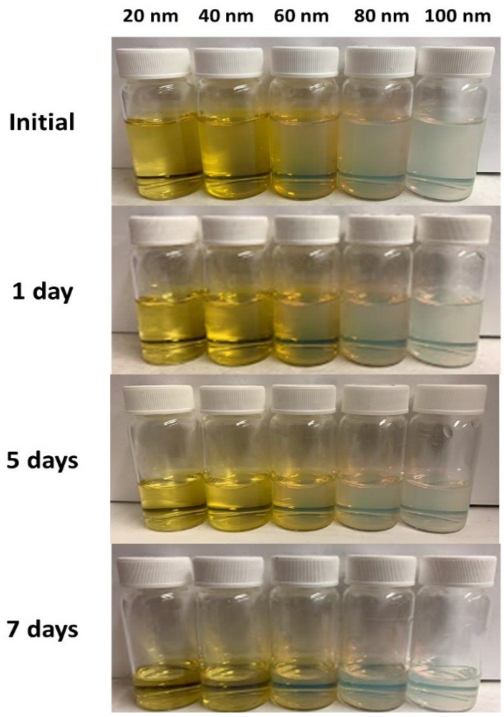

Figure S3. Photographs of the Ag-SOA-PC-HT nanoparticles (6 ppm) after initial synthesis, day 1, 5-days, and 7-days post synthesis in FW at 25 ºC.

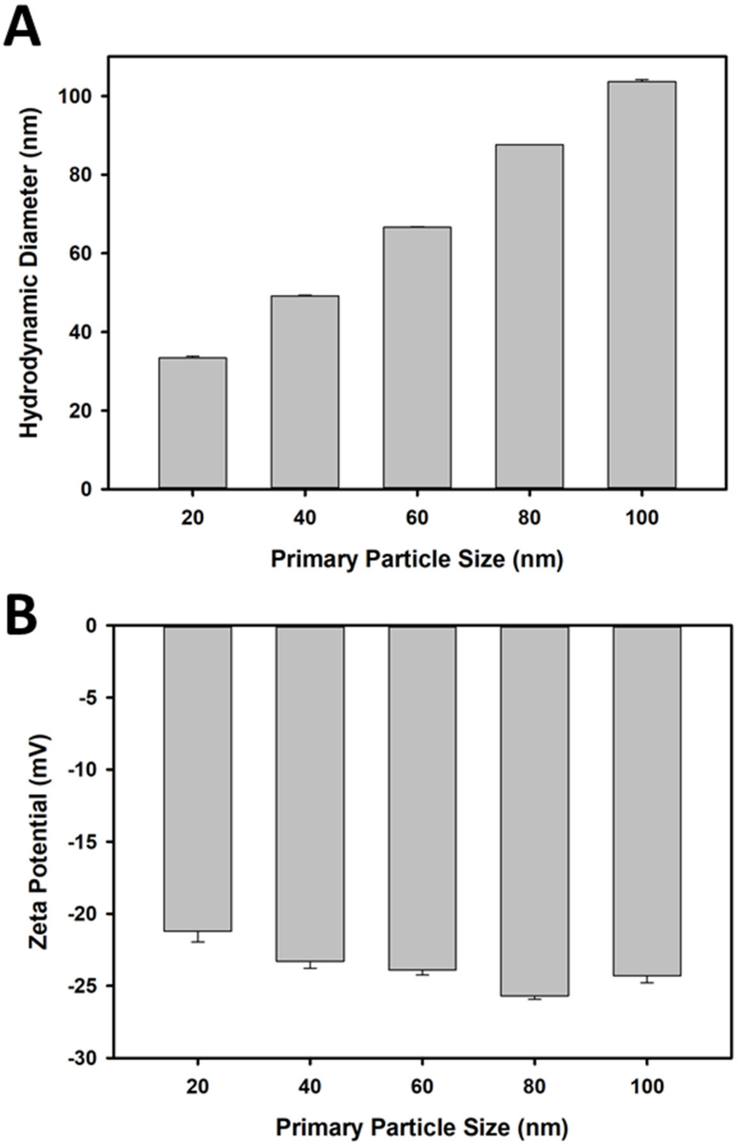

Figure S4. A) Hydrodynamic diameter and **B**) zeta potential of 20, 40, 60, 80 and 100 nm Ag-SOA-PC-HT nanoparticles at 12 mg Ag/L in fishwater.

Table S1. Properties of citrate-capped AgNP properties from manufacturer (NanoComposix, San Diego, CA).

AgNP Size (nm)	HDD (nm)	Zeta (mV)	Particle Concentration (AgNPs/mL)	λ_{max} (nm)	OD_{max}
19.7 ± 3.6	23	-41	$4.9*10^{11}$	392	4.21
41 ± 5	44	-50	$5.4*10^{10}$	417	2.9
59 ± 6	64	-51	$1.8*10^{10}$	438	1.93
80 ± 9	85	-52	$7.3*10^{9}$	463	1.37
97 ± 13	101	-51	$4.2*10^{9}$	485	1.05

Table S2. Ag-SOA-PC-HT concentrations determined by ICP-MS.

AgNP Size (nm)	Particle Concentration (mg Ag/L)	Particle Concentration (AgNPs/mL)
20	32.98	$7.7*10^{11}$
40	37.78	$9.7*10^{10}$
60	41.37	$3.5*10^{10}$
80	42.01	$1.5*10^{9}$
100	59.71	$1.2*10^{10}$

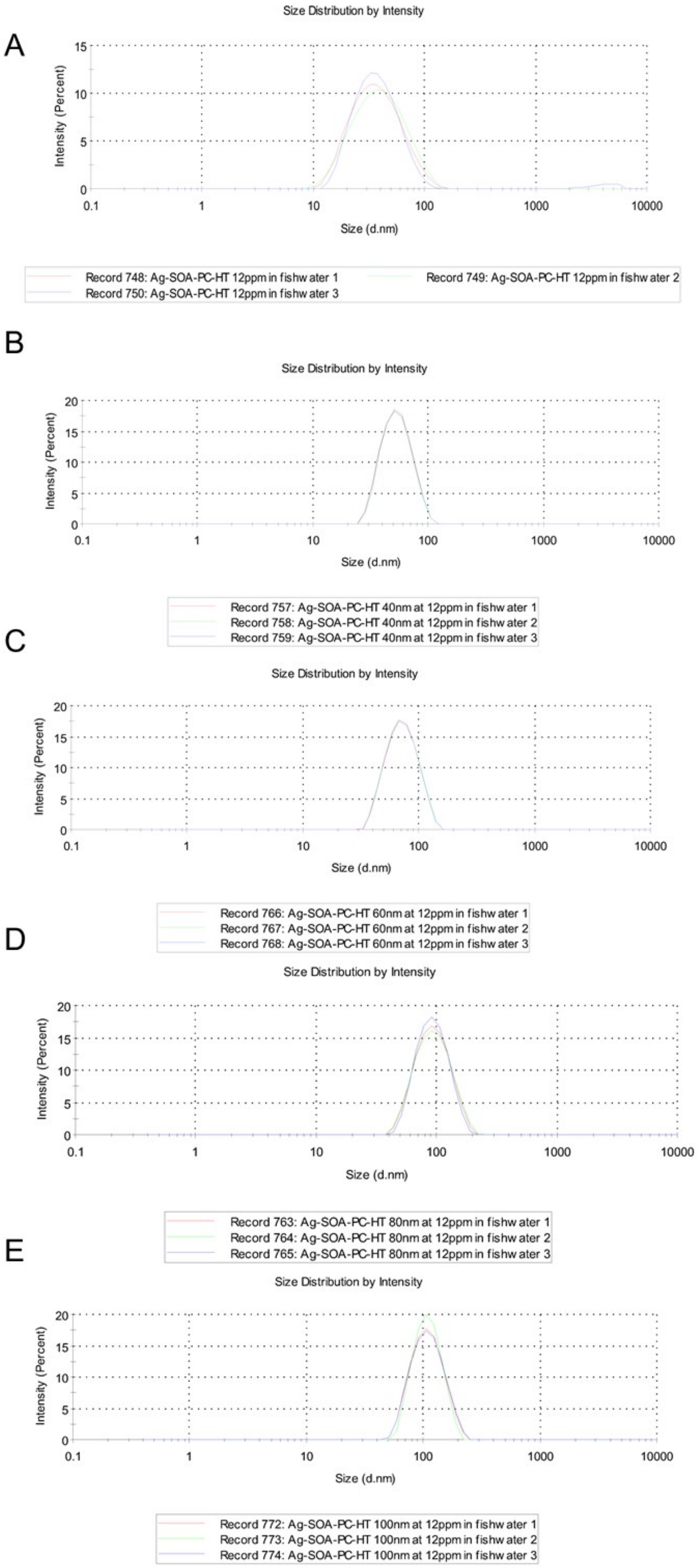

Figure S5. Size distribution by intensity curves for (**A**) 20 nm, (**B**) 40 nm, (**C**) 60 nm, (**D**) 80 nm, and (**E**) 100 nm Ag-SOA-PC-HT NPs.
